# Novel Technologies towards the Implementation and Exploitation of “Green” Wireless Agriculture Sensors

**DOI:** 10.3390/s24113465

**Published:** 2024-05-28

**Authors:** Loukia Vassiliou, Adnan Nadeem, David Chatzichristodoulou, Photos Vryonides, Symeon Nikolaou

**Affiliations:** 1 Electrical Engineering Department, Frederick University, Nicosia 1036, Cyprus; lvassiliou@ari.moa.gov.cy (L.V.); anadeem.msee19rimms@seecs.edu.pk (A.N.); eng.cd@frederick.ac.cy (D.C.); p.vryonides@frederick.ac.cy (P.V.); 2 Agricultural Research Institute, Aglantzia 1516, Cyprus; 3 RF and Microwave Solutions Ltd., Dromolaxia 7020, Cyprus; 4 Frederick Research Center, Nicosia 1036, Cyprus

**Keywords:** precision agriculture, additive manufacturing, humidity sensors, RFID sensors, WPT, UAVs, energy harvesting, beamforming, battery-less wireless sensors

## Abstract

This manuscript presents the use of three novel technologies for the implementation of wireless green battery-less sensors that can be used in agriculture. The three technologies, namely, additive manufacturing, energy harvesting, and wireless power transfer from airborne transmitters carried from UAVs, are considered for smart agriculture applications, and their combined use is demonstrated in a case study experiment. Additive manufacturing is exploited for the implementation of both RFID-based sensors and passive sensors based on humidity-sensitive materials. A number of energy-harvesting systems at UHF and ISM frequencies are presented, which are in the position to power platforms of wireless sensors, including humidity and temperature IC sensors used as agriculture sensors. Finally, in order to provide wireless energy to the soil-based sensors with energy harvesting features, wireless power transfer (WPT) from UAV carried transmitters is utilized. The use of these technologies can facilitate the extensive use and exploitation of battery-less wireless sensors, which are environmentally friendly and, thus, “green”. Additionally, it can potentially drive precision agriculture in the next era through the implementation of a vast network of wireless green sensors which can collect and communicate data to airborne readers so as to support, the Artificial Intelligence and Machine Learning-based decision-making with data.

## 1. Introduction

**Figure 1:**
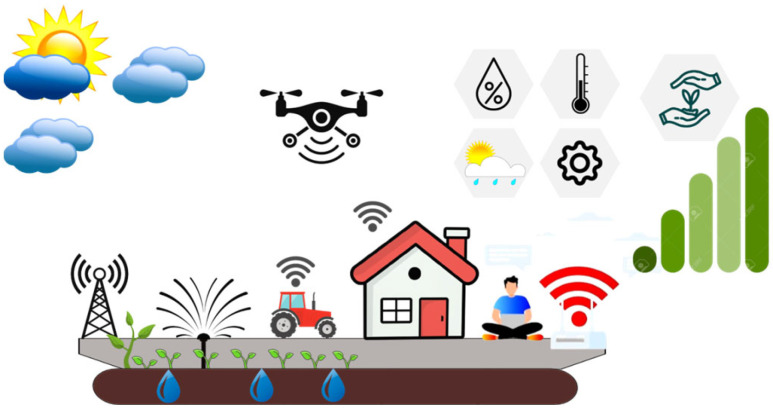
Conceptual diagram of precision agriculture.

The advances in technology and the need for sustainable agricultural practices are driving Precision Agriculture (PA) and Smart Farming (SF) [1] as depicted in Figure 1. The most widely used technologies involve the use of IoT devices, UAVs, AI, and big data analytics [2] to optimize crop production, water and nutrients allocation, and decision-making procedures. By integrating data from various types of sensors, farmers can precisely monitor and manage field conditions, leading to increased yields while ensuring minimized negative environmental impact. The potentials of PA and SF smart farming include improved efficiency, cost savings, and the ability to address global food security challenges through data-driven agricultural practices tailored to local conditions.

The deployment of agriculture wireless sensors [3] has revolutionized farming practices by providing real-time data which are crucial for optimal crop management. The most widely used sensors in agriculture are moisture/humidity [4,5] and temperature sensors [6,7]. Soil moisture sensors aid in precise irrigation scheduling, while temperature sensors help prevent frost damage and optimize growing conditions. Crop health sensors, often utilizing multispectral imaging, enable early detection of pests and diseases. Since many sensors are wireless and are widely scattered in large area fields, their precise location must also be monitored. For wireless IoT sensors [8,9], additive manufacturing [10] can provide an environmentally friendly biodegradable package [11]. In addition, additive manufacturing enables the rapid production of customized housing for wireless agriculture sensors tailored to specific environmental conditions. With 3D printing sensor enclosures, farmers can design shapes optimized for the sensing feature and the deployment conditions. Overall, it streamlines sensor deployment [12], maintenance, and innovation in modern farming practices.

RF energy harvesting circuits [13] capture electromagnetic energy from ambient radio frequency signals, converting it into electrical power to sustain battery-less wireless IoT sensors [14,15]. Designated transmitters utilize the Wireless Power Transfer (WPT) [16] principle and emit radiation which is collected by wireless sensors with an Energy Harvesting feature. This additional feature allows the wireless sensor platforms to eliminate the need for physical connections or batteries. By harvesting RF energy from the surrounding wireless communication networks or designated transmitters, these circuits enable the continuous operation of sensors in various environments. This approach enhances scalability and reliability while reducing maintenance costs and environmental impact, making it ideal for IoT deployments and remote monitoring applications. It is possible for these WPT transmitters to be carried by UAVs [17] in order to secure a short distance between the transmitter and the ground-based sensors. This customized WPT transmitter can be added to already-used UAVs, which are already exploited in PA [18] through high-resolution imaging, that allow farmers to monitor crops with precision and detect issues such as nutrient deficiencies, pest infestations, or irrigation problems. UAVs can swiftly cover large agricultural areas, providing timely data and potentially being able to wirelessly charge a large number of IoT wireless sensors with Energy Harvesting capabilities.

The current manuscript presents methods of utilizing novel technologies such as (a) additive manufacturing, (b) RF energy harvesting, and (c) the use of UAVs as WPT transmitter carriers so as to support the implementation and exploitation of battery-less “green” wireless sensors. RF energy harvesting refers to a feature on the receiver’s side to be able to collect RF energy using an antenna, convert it to DC, and use it at will. WPT refers to the use of a designated transmitter in order to wirelessly charge receivers with the Energy Harvesting feature.

This manuscript is organized into three technical sections, and Section 2, Section 3 and Section 4 describe specific uses of the aforementioned technologies. More specifically, Section 2 discusses the use of additively manufactured passive humidity sensors; Section 3 discusses the addition of the EH feature on dual frequency receivers using either the UHF or the ISM band; and Section 3 discusses an experimental setup for a UAV-carried transmitter that is used to wirelessly charge (WPT) ground-based sensors. Finally, Section 5 provides the conclusion on the possible uses of the discussed technologies in Precision Agriculture.

## 2. Additively Manufactured Passive Agriculture Sensors for Humidity Sensing

Soil humidity is one of the most important parameters that needs to be monitored and controlled for most agriculture crops. Therefore, the implementation of soil moisture sensors was set as one of the primary objectives of the ICARUS project. The use of additive manufacturing for the implementation of battery-less “green” sensors led to the design and manufacturing of passive humidity sensors where the sensing principle is based on the frequency shift that is caused by the wireless sensor’s antenna as a result of the humidity variation. Two solutions were investigated. The first one was the use of a compact dipole antenna at 3.5 GHz, where the humidity-sensitive PEDOT: PSS material was used, and the second one was the use of a UHF RFID antenna embedded in soil, which exploited the effective dielectric constant change, associated with the surrounding soil’s moisture.

### 2.1. A 3.5 GHz 3D-Printed Dipole

**Figure 2:**
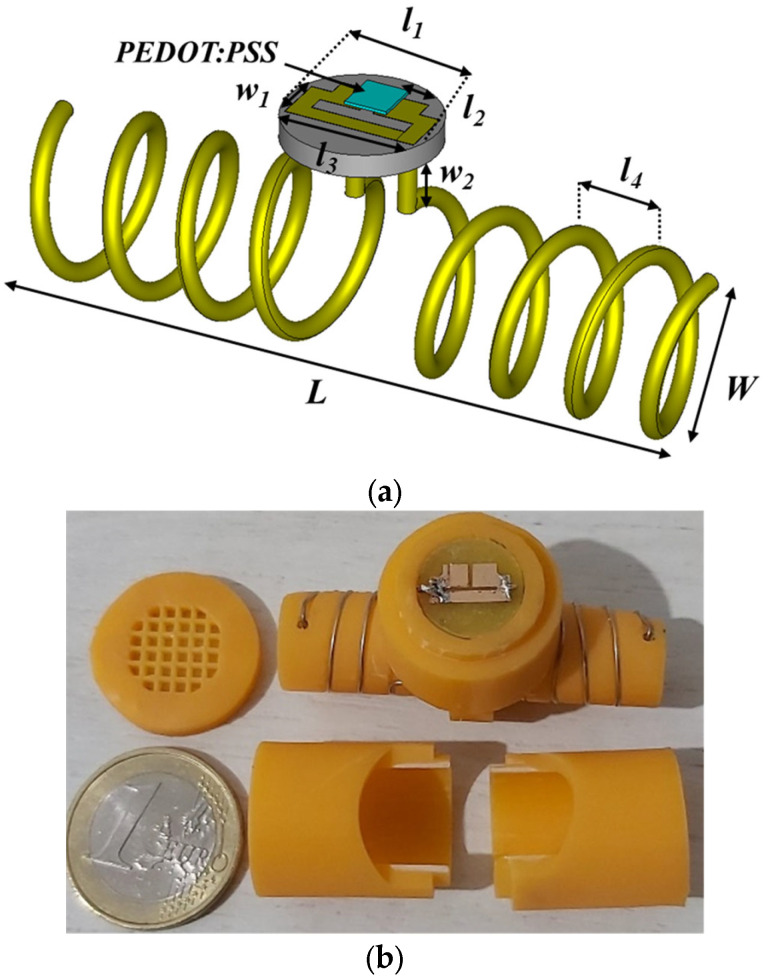
3D-printed dipole for humidity sensing. Dimensions (mm): W = 15.1, L = 68, l_1_ = 10, l_2_ = 3, l_3_ = 9, l_4_ = 8.5, w_1_ = 3, w_2_ = 5, N = 3.5.

The implemented dipole [19] consists of two metallic spirals and the termination load consisting of an inkjet-printed PEDOT: PSS material which is sensitive to humidity changes. 3D printing was used to implement the carriers of the metallic spirals and the sensing material cage that was porous in order to allow for the penetration of the soil in the sensing material compartment. Figure 2 indicates the dipole’s geometric characteristics. The PEDOT: PSS block implements a termination load for the dipole, and its effective impedance changes with different humidity levels. As a result, the effective resonance of the 3.5 GHz dipole shifts for different humidity levels, and therefore the humidity can be indirectly identified. Table 1 presents the association of the effective εr with the recorded and verified moisture percentage for two frequencies: the introduced one at 3.5 GHz, and the subsequent case where a UHF dipole at 915 GHz is used instead.

**Figure 3:**
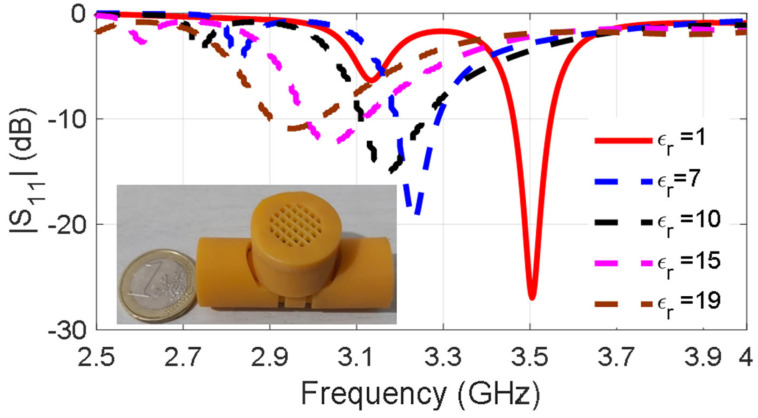
Self-resonance shifts in the presence of varying moisture soil.

Figure 3 presents the frequency shifts of the 3D-printed dipole associated with the varying dielectric constant which is the result of different humidity levels. Despite the simplicity of the passive humidity sensing, there are two important drawbacks that limit its applicability and usefulness. The first one is based on the repeatability and reliability of the used method. Effective εr can be affected by other parameters as well, such as the salinity of minerals in the soil, and as a result, the detected frequency shifts cannot be exclusively associated with humidity changes. This can lead to inaccurate humidity readings. The second one is associated with the operation frequency. At 3.5 GHz, the propagation in soil is very lossy, and, consequently, the reading range of the wireless sensors is limited. In order to overcome the first drawback, the use of a more reliable IC-based digital sensor was preferred, and it is discussed in Section 3. To overcome the read range limitations, sensors operating at lower frequencies (under 1 GHz) were used.

### 2.2. UHF 3D-Printed Dipole

**Figure 4:**
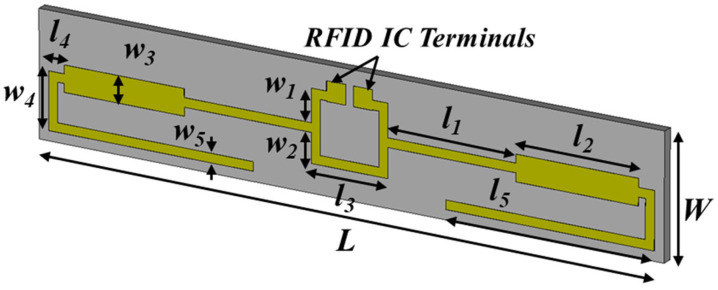
RFID dipole for humidity sensing. Dimensions (mm): W = 16, L = 96, l_1_ = 20, l_2_ = 19, l_3_ = 1_2_, l_4_ = 2.5, l_5_ = 31, w_1_ = 13.6, w_2_ = 2.5, w_3_ = 2.4, w_5_ = 6.

**Figure 5:**
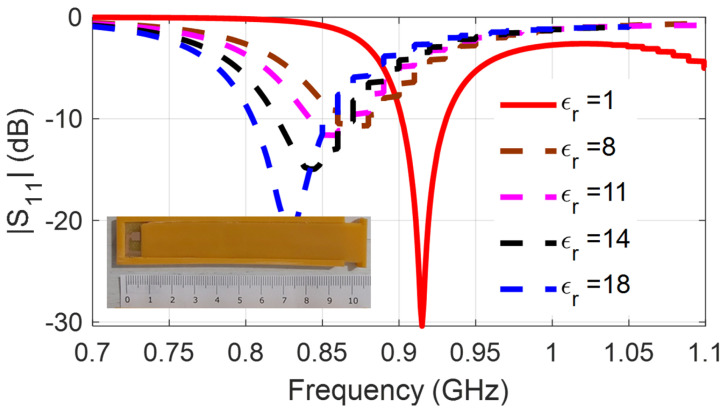
Self-resonance shifts in the presence of varying moisture soil.

For the implementation of additively manufactured RFID-based sensors [19], a paper substrate and an inkjet-printed conductive nano-particle ink were used. The printed planar-folded dipole is depicted in Figure 4, and a 3D-printed protective cover is used to protect the sensitive paper substrate. The capacitive load of the IC has impedance 13.3-j122, and the folded arms of the dipole add inductive load for the conjugate matching. Self-resonance in free space occurs at 915 MHz and when the sensor is embedded in soil, the self-resonance shifts, as can be seen in Figure 5. The higher the effective dielectric constant, the lower the self-resonance occurs. The association of the effective εr with the moisture percentage has been presented in Table 1. Although the read range is improved as a result of the lower propagation loss at the UHF frequency, the reliability and repeatability issues for the readings remain, and this is why the use of IC humidity sensors has been preferred. To achieve high radiation efficiency, UHF radiators need to be relatively large (in the order of half-wavelength). Therefore, part of the research effort was focused on the miniaturization of the antennas of the wireless sensors.

## 3. Agriculture Sensors with Energy Harvesting Feature

**Figure 6:**
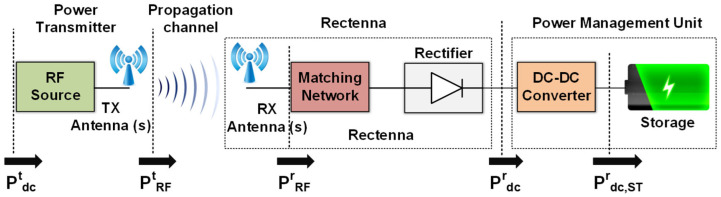
Wireless Power Transfer to a sensor with the Energy Harvesting feature.

For the extensive use of wireless sensors which are needed for Precision Agriculture, battery-less sensors should be used to avoid the cost and time consumption from battery replacement and possible soil contamination from the batteries’ chemicals. PV cells are often used to power agriculture sensors, but this requires a large-sized sensor, and a large area and high-cost photovoltaic cell. It also requires direct exposure to the sun, and so, in other words, it cannot be used for sensors which are implanted in soil. An alternative possibility is the use of RF Energy Harvesting. Any radiation transmitter that is used for communication purposes can be also used as a potential source of RF power. An antenna, in combination with a rectifier (rectenna), can collect this RF power and convert it to DC power. This harvested energy can be used to power low-power sensors which act as transmitters for a short period of time, usually when interrogated by a designated reader, while they can remain in “listening mode” for a much longer period of time. During this period of time, they can harvest ambient RF power, or they can receive purposely transmitted wireless power in a designated Wireless Power Transfer (WPT) scheme so as to power the associated sensor. If the real-time converted power is insufficient for the needs of the sensors, a power management unit is also required. The concept of the harvested energy in a wireless power transfer scheme is presented schematically in Figure 6. Depending on the implementation, the wireless sensor may use a common antenna for the communication and for the Energy Harvesting system, or it may more often use dual antennas having the first designated for the communication system and the second for the Energy Harvesting system. In the current section, two implementations which use the UHF and ISM bands are presented.

### 3.1. ISM Sensor Platform with an Integrated UHF Energy Harvesting Feature

**Figure 7:**
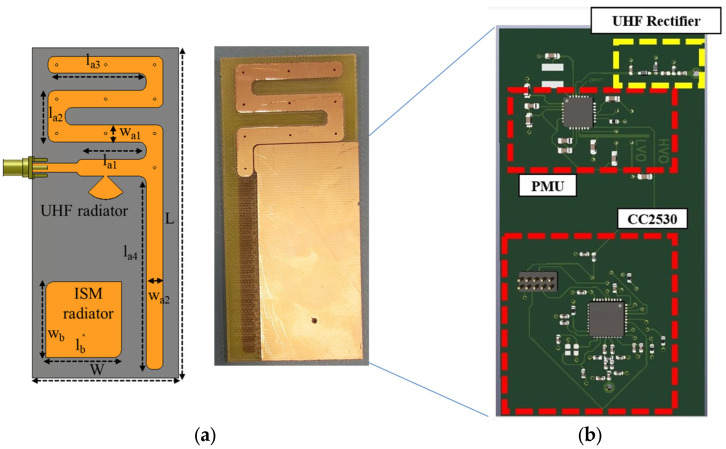
(**a**) Schematic of the modules’ antennas. Dimensions (mm): L = 100, W = 47, la_1_ = 18.4, la_2_ = 16.5, la_3_ = 37.5, la_4_ = 62, lb = 24.5, lg = 75, wa_1_ = 18.4, wa_2_ = 18.4, wb = 24.5, wg = 35. (**b**) Prototype with the embedded circuit’s implementation with the rectifier, the PMU, and the communication module in a single board.

The dual-frequency system uses a meander UHF antenna [20] integrated with a lumped elements rectifier and a power management unit (PMU) based on the e-peas AEM30940 IC. For the communication module, a truncated-corners patch antenna with circular polarization is used which is connected to the TI CC2530 radio transceiver, integrating an Intel 8051 MCU, which adopts a protocol for communication named Simplicity. The TI-developed module is suitable for integration with a variety of digital sensors, including temperature and humidity sensors. The implemented wireless sensors platform with the integrated Energy Harvesting feature is presented in Figure 7.

**Figure 8:**
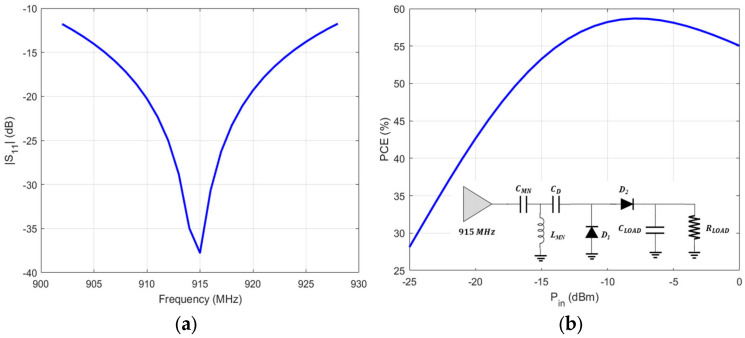
Lumped elements rectifier with insertion loss (|S_11_|) and efficiency (PEC) plots.

A UHF meander monopole is used so as to reduce the overall size of the antenna. A radial stub is used to achieve the matching with the lumped elements rectifier. In order to avoid the large area required when distributed elements are used, lumped components are utilized instead. The equivalent circuit of the rectifier, with its return loss, and efficiency plots are presented in Figure 8. The effective RF-to-DC efficiency is defined as the ratio of the RF power at the input of the rectifier and the DC power on the ohmic load (R_L_) that terminates the non-linear rectifier, as given in Equations (1)–(2). The RF power at the input of the rectifier is measured using a spectrum analyzer attached to the antenna that is connected with the rectifier, and the DC power on the load is calculated by measuring the DC voltage on the load using a digital multi-meter.

(1) $$\eta =P_{RF}/P_{L}$$

(2) $$P_{L}=V^{2}/R$$

The implemented rectifier is composed of a lumped input matching network (an RF capacitor CMN1 = 0.5 pF, and a shorted RF inductor in parallel LMN = 23 nH), two Schottky diodes D_1_ and D_2_ (Skyworks SMS7630-079LF), two RF capacitors at 1 nF (CD = CLOAD). The termination resistive load is 12.9 KΩ (RLOAD) and is setting the cut-off frequency of the output low-pass filter before the input stage of the subsequent PMU module.

For the implementation of the PMU e-peas AEM30940 IC has been selected. This IC can support a cold start from 380 mV which can be activated for a minimum input power equal to –22 dBm. After the cold start, assuming that the input voltage is between 50 mV and 5 V, the PMU-integrated boost converter can extract the available power from the source. This performance makes the specific lumped elements rectifier operational for even lower input power that can be as low as –32 dBm. This proposed PMU IC is able to supply low-voltages (up to 1.8 V) and typically drive a low-power microcontroller unit (MCU) like the used TI CC2530 IC, which is powered without the need of a battery source, providing a battery-less platform for agriculture sensors.

### 3.2. UHF Sensor Platform with an Integrated ISM Energy Harvesting Feature

**Figure 9:**
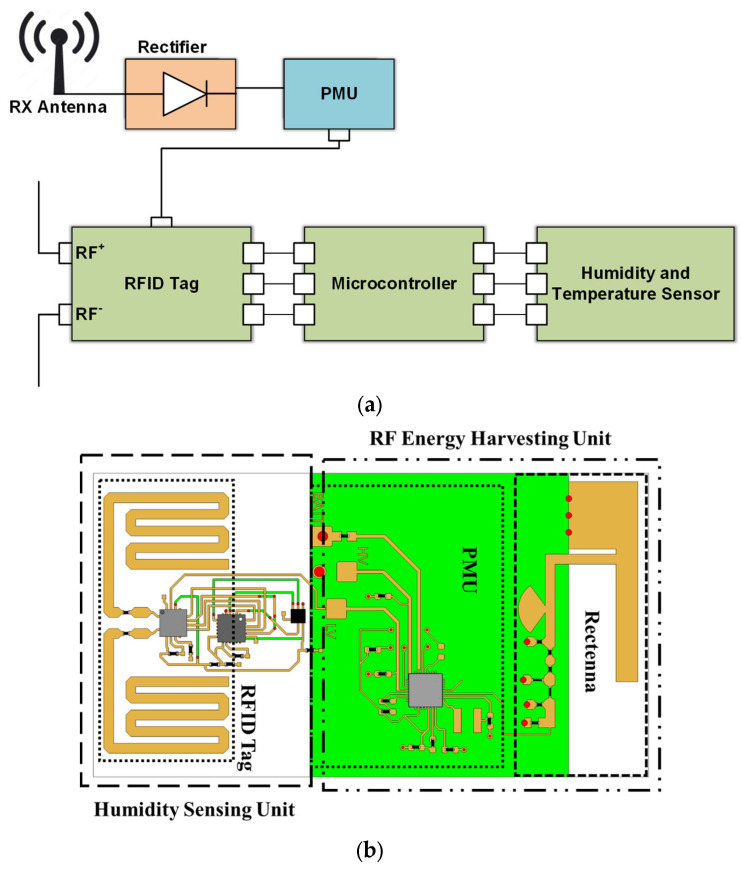
Block diagram and IC layout of the implemented module.

An IC sensor for temperature and humidity-sensing capabilities is integrated into a similar platform where the UHF band is used for the communication module and the ISM band for the Energy Harvesting [21]. A PIFA antenna and a series rectifier operating at 2.45 GHz (ISM band) implement the Energy Harvesting unit that, in combination with the PMU, replaces the need for a battery. The PMU based on the e-peas AEM30940 IC provides a regulated 1.8 V DC voltage to the RFID tag IC (ROCKY100), assuming that the input RF power is higher than −22 dBm. For the sensing information communication, a UHF meander dipole antenna is used operating at 915 MHz. When the regulated 1.8 V is supplied from the PMU, the tag IC operates in semi-passive mode, and it effectively increases its read range. The used RFID IC is ROCKY100, which is EPC C1G2 compliant and is compatible with the power harvesting modules and SPI communication. This makes it compatible with the PMU and the HTS221 humidity and temperature IC sensor, which can be used for agriculture applications that involve soil measurements. The conceptual module and the IC layout of the proposed UHF platform with the integrated Energy Harvesting feature can be seen in Figure 9. The process of measuring the relative humidity and temperature is controlled with a Texas instrument mixed-signal microcontroller including two SPI interfaces to communicate with the RFID IC. Upon receiving an SPI-directed read request from the RFID reader, the ROCKY100 SPI bridge requests the value of the last measurement. Then, the recorded reading from the HTS221 IC is sent to the RFID IC, and from there to the RFID reader. The use of the integrated Energy Harvesting feature results in the enhancement of the read range of the wireless sensor and a battery-less, and thus “Green”, operation.

**Figure 10:**
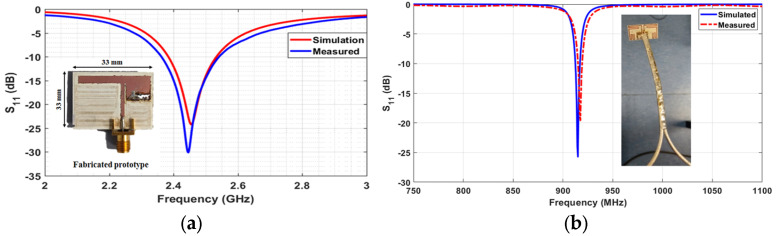
The PIFA antenna for the Energy Harvesting ISM module, and the meander monopole for the communication UHF module.

The commercially available HTS221 is a sensor for relative humidity and temperature sensing that includes a sensing element and can operate over a temperature range of −40 °C to +120 °C. The sensor IC requires a low supply voltage that can be covered from the low-voltage output terminal of the PMU, which is regulated at 1.8 V. The sensing element is integrated with an IC able to communicate the sensed information in the form of a digital signal to the microcontroller through the I²C/SPI (Inter-Integrated Circuit)/(Serial port interface) interface. Considering that a commercially available temperature and humidity IC sensor is used, emphasis is given to the implementation of the Energy Harvesting module and the UHF communication antenna. The two antennas, are presented in Figure 10 with their measured insertion loss.

**Figure 11:**
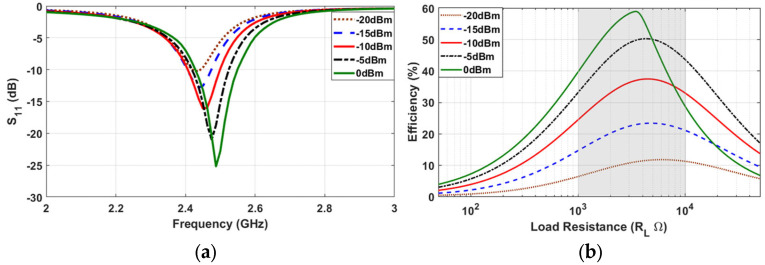
Matching and efficiency of the implemented voltage doubler rectifier.

A voltage-doubler topology is used as a rectifier. Despite its inherent non-linear nature, it is well-matched for different levels of input power ranging from −20 dBm to 0 dBm, as can be seen in the response in Figure 11. The effective RF-to-DC efficiency depends heavily on the input power, and it has a peak value at almost 60% for input power at 0 dBm (Figure 11).

The presented battery-less RFID-based humidity and temperature sensor with the integrated Energy Harvesting feature presents a “green” solution for agriculture applications. The sensing module utilizes the harvested power from the PMU to power up the ROCKY100 RFID IC. With the provided power, the IC operates in semi-passive mode, and it significantly increases its read range. The sensing information is communicated as a digital signal from the digital capacitive HTS221 humidity sensor using the SPI and I2C interface pins, and the data are transferred to the RFID IC through the MSP430 ultra-low-power microcontroller. When an interrogation read command is received, the sensor data are transmitted to the RFID reader, along with the RFID tag ID, through the RFID UHF meander dipole.

## 4. Wireless Power Transfer from UAV-Carried Transmitters to Ground-Based Sensors

The Energy Harvesting feature of the green wireless sensor platform is utilized when a transmitter is designated with the wireless charging of the sensors. This can be accomplished with Wireless Power Transfer, which is the radiation of un-modulated waves (power) directed in high-gain beams aiming to increase the power density at the receivers. Considering the free-space losses, it is often critical to maintain a small distance between the transmitter and the receiver modules. Carrying the transmitter on a flying UAV provides some controlof the distance between a mobile transmitter and a stationary ground-based receiver.

### 4.1. WPT at UHF Frequency–915 MHz

**Figure 12:**
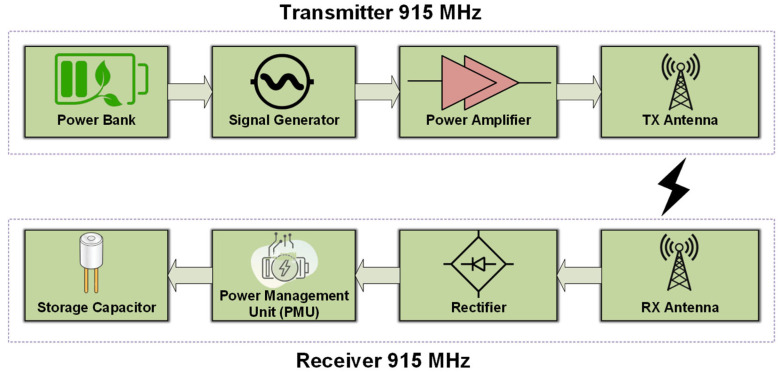
WPT experiment—TX and RX building blocks.

Wireless charging from a UAV-carried transmitter towards ground-based sensor platforms with the Energy Harvesting feature has been tested. In the described experiment, the block diagrams of both the transmitter and receiver modules are presented in Figure 12. The ground-based sensors use the UHF Energy Harvesting system that was described in Section 3.1 with a resonance frequency of 915 MHz (Figure 8). During the experiment, which took place in an open field, the UAV s hovered at a height of 1.6 m above the ground and the ground-based sensors were positioned in one of the 25 positions of the grid (5 × 5). During the WPT process, a direct line of sight (LOS) was ensured, and the voltage of the capacitor was monitored until it reached the maximum possible voltage at 3.5 V, which is equivalent to the maximum stored energy for the Energy Harvesting system. The measurement across the capacitor was repeated 25 times, one for every possible position of the sensor on the grid. Three different ground-based sensors were used, and the measurements implied that their charging was uncorrelated, indicating that the charging of multiple sensors can take place simultaneously.

For the experimental setup of wireless power transfer from a UAV-carried Transmitter (TX) to the ground-based sensors, a frequency of 915 MHz (UHF) was used over the ISM so as to limit the free-space losses. In order to integrate the entire TX system on a flying UAV, a portable signal generator was used that produces 0 dBm output power at 915 MHz and was powered by a light-weight power bank. For the purpose of boosting the TX power, a power amplifier at 915 MHz was utilized that had 30 dB gain. For the transmitter antenna, a compact patch with gain 2 dBi was used.

On the receiver (RX) side, the compact circularly polarized antenna with gain 2 dBi was used in combination with a voltage doubler rectifier at 915 MHz. The rectified voltage was stored in a commercially available Power Management Unit (PMU) with a 4.7 mF capacitor as the power storage element.

**Figure 13:**
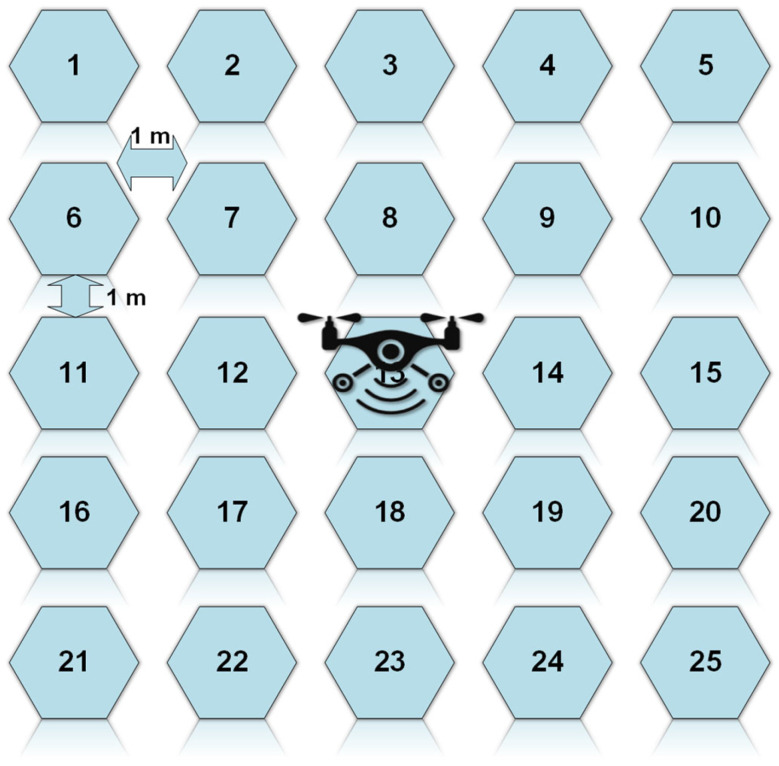
A 5 × 5 square grid for the experimental setup.

For the experimental setup, a UAV with the transmitter hovered at a height of 1.6 m from the ground. One Energy Harvesting receiver (Figure 11) was placed on the surface of the ground in each of the 25 grid points (Figure 13), and the voltage on the 4.7 mF storage capacitor was monitored until it reached the maximum output voltage from the PMU. For the measurements, a direct line of sight (LOS) between the transmitter and the receivers was ensured.

**Figure 14:**
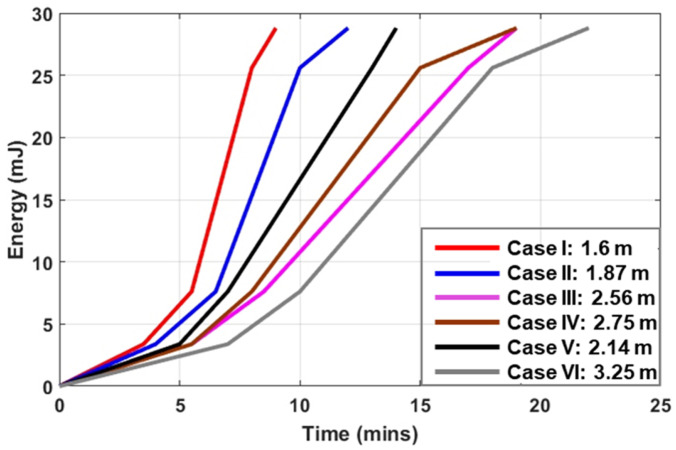
Charging time for a 4.7 mF capacitor to reach the maximum stored energy of 28.8 mJ (under the maximum voltage of 3.5 V) for different distances between the transmitter and the Energy Harvesting system.

There are six cases presented in Table 2, where the Energy Harvesting receiver was placed at six different positions which are numbered on the rectangular grid on the ground. For each case and considering that the UAV was hovering at a fixed position of 1.6 m over square 13, the elevation and azimuthal angles are marked. The charging times which are recorded indicate the time that is needed, under the aforementioned conditions, for the 4.7 mF storage capacitor to reach 1.2 V, 1.8 V, 3.3 V, and 3.5 V, respectively. These voltage levels are associated with the output voltage, which can be provided by the PMU. According to the PMU specifications, the maximum voltage that can be supplied to the storage element is 4 V. The storage capacitor provides the necessary power to the sensing board that is connected to the PMU when there is no RF power available. The graphs of all six cases of energy storage (mJ) in a charging capacitor versus charging time (mins) are plotted in Figure 14. Expectedly, the closer a rectenna is the higher level of power it receives and, consequently, the faster the charging element acquires sufficient energy to power the communication module. In conclusion, the experimental setup indicated that the implemented Energy Harvesting module can provide sufficient power to allow for the battery-less operation of the used sensors, and for the given transmitter specifications, the RF energy harvesting was possible for a maximum distance of 18 m under optimum antenna orientation and polarization conditions. The duration of the WPT process depends on the flight duration and the flight altitude of the UAV that carries the UHF transmitter.

### 4.2. Communication Range between the UAV-Carried RFID Reader and the UHF Communication Module

**Figure 15:**
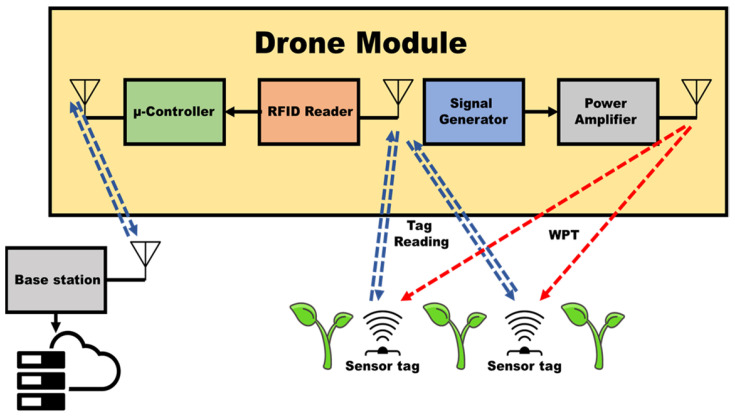
Communication module consisting of an RFID reader and WPT transmitter.

The block diagram of the UAV-carried communication system is presented in Figure 15. The communication module consists of three sub-systems. The WPT system has been described in the previous section. There is also a portable UAV reader for the communication with the RFID-enabled sensors, as well as a module that communicates with a base station over WiFi. This is implemented using an Arduino UNO microcontroller board with an ESP8266 Wi-Fi communication module.

Based on the power consumption data in Table 3, which summarize the power consumption of the individual sensing components, the entire sensing module consumes around 0.5 mW that can be easily provided by the PMU storage capacitor, which can store up to 28.8 mJ of energy. This power can be provided for sufficient time for tens of read cycles from the RFID reader. The charging time depends upon the receiver’s distance from the transmitter. As per the measured data extracted from Table 2, if the receiver is placed 1.6–1.87 m away from the transmitter, it will take approximately one minute for the PMU to charge the capacitor and be in a position to provide 0.5 mW to the sensing module for its operation. Similarly, if the receiver is placed 2–2.5 m far from the WPT transmitter, it will need approximately 2 min for the PMU to get charged and be able to provide 0.5 mW to the sensing module. Assuming a fully charged capacitor (28.8 mJ) and considering that a read cycle from any RFID reader takes less than 1 s, there is sufficient power for more than 60 read cycles before the EH system needs to be charged again.

For the conducted experiment, which took place in laboratory environment in order to control the orientation of the transmitter and receiver antennas, the UHF Fonkan reader used a transmission (TX) power of 23 dBm, while the gain of the transmitter antenna was 2 dBi. Under these conditions, the maximum read range of the passive tag was up to 2 m, and in the semi-passive or WPT-enabled mode, the best-case scenario read range was almost 5 m. When a power amplifier was added and a higher gain (7 dBi) antenna was used as the transmitter antenna, the transmitter power rose to 33 dBm, the read range of the passive UHF tag with the ROCKY100 RFID IC was tested up to 11 m, and for the semi-passive tag with the Energy Harvesting feature that was described in Section 3.2, the read range was 25 m (under optimal antenna orientation and polarization conditions).

The RFID tag read range was enhanced by operating the tag in semi-passive mode when the required DC power was provided from the RF Energy Harvesting module that was integrated with the humidity sensors. For the on-field testing, a customized UAV-carried reader and the customized sensors were used. Each sensing module consumes approximately 0.5 mW of power. This power can be provided for sufficient time from the energy that is stored in the storage capacitor (~28.8 mJ) assuming that the high voltage terminal of the PMU is used. The point-to-point communication between two microcontrollers (one deployed on the flying UAV and another at the base station) is effective up to 50 m using an omni-directional antenna at the base station. The maximum reading distance of the UAV reader depends on the EIRP. For the maximum transmitted power at +33 dBm and transmitter antenna gain at 7 dBi, the maximum reading range was 25 m for the semi-passive tag and 11 m for the tag operating in the passive mode. The RFID reader can simultaneously read more than 20 sensors scattered in an open field. The flying time of the UAV depends on the external load and the size of the battery.

The on-field testing demonstrated the feasibility of WPT from a UAV to wireless sensors with the Energy Harvesting feature. The conducted experiment demonstrated the effectiveness and the usefulness of using UAV-carried readers to collect data from battery-less “green” agriculture sensors.

### 4.3. UAV Readers with Beam-Forming Capability

**Figure 16:**
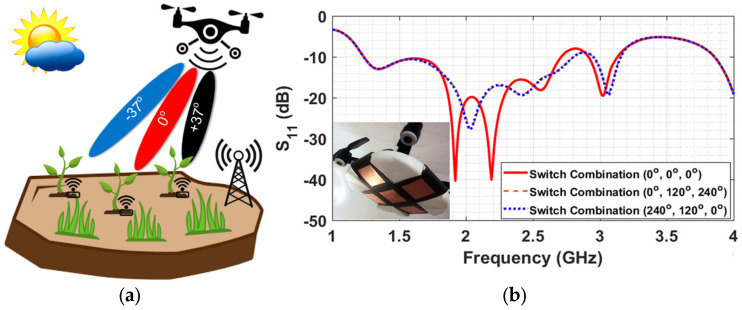
Beam-forming concept, and the insertion loss of the prototype cross-shaped array at 2.4 GHz.

The experiments indicated that using high-gain antennas can significantly increase the charging and the reading distances and can also support the simultaneous charging of several wireless sensor platforms with the Energy Harvesting feature. This observation led to the design of a UAV-carried beam-forming array which can direct the maximum gain of the radiation beam in five different pre-determined directions along two perpendicular planes [22]. The concept idea and a prototype conformal cross-shaped array designed for the ISM band at 2.4 GHz are presented in Figure 16. The schematic in Figure 16a demonstrates the concept of using beam-forming in order to increase the gain in the desired directions and eventually the communication (or power transfer) range between the UAV-carried transmitter and the ground-based sensors. Figure 16b shows the insertion loss of the conformal linear arrays when the maximum gain is directed in one of the three directions (0° and +/−37°). The three directions are achieved when the phase shifter causes a phase difference on the radiation elements (0° or + 120° or + 120°) and the plots for the two symmetric cases (+/−120°) coincide. In the inset, the prototype of the implemented orthogonal 3 × 1 linear array is presented.

**Figure 17:**
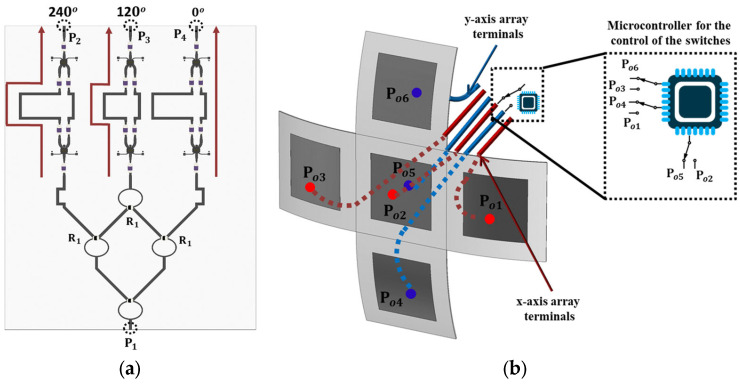
(**a**) Feeding Network consists of 1:2:1 power divider followed by a three-stage phase shifter (**b**) switching between the two orthogonal 3 × 1 linear arrays using SPDT switches controlled with a Microcontroller.

For the prototype implementation, the ISM band was preferred because the two 3 × 1 linear arrays of patch radiators would be much bigger if the UHF band was used instead. In addition, the use of a beam-forming array is more useful for radiation in the ISM band because of the higher free-space losses that ISM frequencies suffer compared to the UHF band. Beam-forming is supported using a reconfigurable phase shifter in conjunction with a power combiner. For the three radiating elements in each of the two 3 × 1 arrays, the power ratios at 1:2:1 and the phase difference of the three adjacent driving currents can be either 0° or +120° or −120°. The power ratio is accomplished using the power divider presented in Figure 17a, which is a combination of three 3 dB Wilkinson dividers [22] arranged so they can have one input port and three output ports. The central port is directed on the central (common) patch element. The 1:2:1 power divider is followed by a three-stage phase shifter. The phase shifter is implemented using two different electric paths per branch, which are selected in combination with using three pairs of single-pole double-through (SPDT) switches [22]. The phase shifter schematic in Figure 15 shows the case when the three patch radiators are fed with 120° phase difference, causing the directivity of the 3 × 1 elements array to form a −37° angle. The set of six SPDT switches can be used in three combinations, and thus the main beam can be directed underneath the UAV to form a 37° angle with the vertical axis, either to the left or to the right. The maximum gain for the 3 × 1 array is 9 dBi and, under optimum orientation conditions, can quadruple the reading range between the UAV-carried reader and a ground-based sensor. In order to switch the feeding from one 3 × 1 array to the other, another three SPDT switches are used, as can be seen in Figure 17b. A switch is needed for the central patch because the feeding point along the patch is different in the two cases so as to maintain the matching. The patch arrays were implemented on a conformal substrate that was formed on the side of the 30 cm radius cylinder, and a Styrofoam supporting structure was implemented for maintaining the aforementioned shape.

## 5. Conclusions

The manuscript provides a comprehensive report of the research findings of the research project ICARUS [23], which investigated the use of additive manufacturing, energy harvesting, and the use of UAVs as carriers of Wireless Power Transfer transmitters and their implementations as part of Precision Agriculture applications. 3D printing and inkjet printing were demonstrated for the implementation of passive humidity sensors. Based on the accuracy and repeatability limitations, the use of commercially available humidity and temperature IC sensors was considered. The IC sensors were integrated on sensor platforms that were using emerging harvesting circuits. Both UHF and ISM bands were utilized for communication and the energy harvesting module. It was demonstrated that a semi-passive RFID-based humidity sensor could quadruple its read range (+6 dB) when it was powered using harvested RF energy collected with the Energy Harvesting system integrated in the sensor’s platform.

Finally, an experimental setup was described which used a UAV-carried WPT transmitter to charge a 4.7 mF capacitor used to store the harvested energy from a ground-based sensor platform. The efficient and effective charging of the customized Energy Harvesting system was demonstrated for distances up to several meters. Wireless charging was demonstrated with uni-directional antennas on the transmitter and the receiver for a distance up to 5 m, and, under optimal orientation and polarization conditions, it could be supported for a distance up to 18 m. The harvested energy was used to power the RFID IC that was able to increase its read range from 9 m to 25 m, proving that UAVs can be used to collect data and power RFID-based ground sensors suitable for Precision Agriculture applications.

## Figures and Tables

**Table 1:** The real part of the permittivity and loss tangent associated with the verified humidity percentage.

For 3.5 GHz								
εr	2	4	7	8	10	12	15	19
tanδ	0.05	0.13	0.15	0.19	0.22	0.25	0.28	0.31
% of moisture	0	5	10	13	15	17	20	25
**For 915 MHz**								
**εr**	**2**	**5**	**8**	**9**	**11**	**14**	**18**	**22**
tanδ	0.08	0.16	0.22	0.25	0.30	0.31	0.36	0.4
% of moisture	0	5	10	13	15	17	20	25

**Table 2:** Charging time for ground-based sensors with the EH system, which are physically located at various distances and in different angles compared to the UAV-carried WPT transmitter.

Grid No.	Distance (m)	θ_angle_ (degree)	φ_angle_ (deg)	Charging Time (mins)			
				(1.2 V) 3.38 mJ	(1.8 V) 7.61 mJ	(3.3 V) 25.6 mJ	(3.5 V) 28.8 mJ
13	1.6	0	0	3.5	5.5	8	9
14	1.87	0	30	4	6.5	10	12
15	2.56	0	50	5.5	8.5	17	19
10	2.75	25	55	5.5	8	15	19
9	2.14	45	40	5	7	13	14
5	3.25	45	60	7	10	18	22

**Table 3:** Power consumption of the sensing module.

Components	Power Consumption	
	Consumed Power	Unit
RFID IC ROCKY100	12.6	μW
MCU MSP430FR2355	468.6	μW
Humidity and Temperature Sensor SHT40	1.6	μW

## Data Availability

The data presented in this study are available on request from the corresponding author.
